# Draft Genome Sequencing of Pseudoalteromonas tetraodonis Strain kknpp56, a Potent Biofilm-Forming Bacterium Isolated from Early-Stage Marine Biofilm

**DOI:** 10.1128/MRA.00605-21

**Published:** 2021-09-23

**Authors:** Meora Rajeev, T. J. Sushmitha, Subba Rao Toleti, Shunmugiah Karutha Pandian

**Affiliations:** a Department of Biotechnology, Alagappa University, Karaikudi, Tamil Nadu, India; b Water and Steam Chemistry Division, Bhabha Atomic Research Centre Facilities, Kalpakkam, Tamil Nadu, India; Montana State University

## Abstract

Pseudoalteromonas tetraodonis strain kknpp56 is an exopolysaccharide (EPS)-producing marine bacterium that forms potent biofilm. To determine the biosynthesis pathways involved in the EPS production of this bacterium, whole-genome sequencing was performed. The complete genome comes from one chromosome containing 3.72 Mbp of DNA with a G+C content of 41%.

## ANNOUNCEMENT

Members of the genus *Pseudoalteromonas* are rod-shaped, Gram-negative, aerobic, heterotrophic bacteria. They belong to the *Alteromonadaceae* family within the *Gammaproteobacteria* class and are mostly distributed in hyper-saline environments, including seawater, sediment, and marine biofilms (1, 2). Members of this genus produce several bioactive compounds that prevent settling of micro- and macrofoulers during biofilm formation in marine environments (3–5). In addition, some species of this genus, *viz.*, *P. ruthenica* and *P. espejiana*, have been shown to produce dense exopolysaccharide (EPS) that helps them in forming potent biofilms on artificial surfaces (6–8).

Here, we report the whole-genome sequence of *P. tetraodonis* strain kknpp56 (GenBank accession number MZ357110). This bacterium was originally isolated from early-stage (72-h-old) marine biofilms assembled in the intake area of a nuclear power plant (08°10′N, 77°42′E) located in the southern coastal region of India (7). Preliminary studies conducted in our laboratory suggested that this bacterium produces dense EPS, which supports it in establishing robust biofilm on artificial surfaces. To understand the biosynthesis pathways involved in EPS production, whole-genome sequencing of *P. tetraodonis* strain kknpp56 was performed using Illumina tag sequencing.

The axenic culture of strain kknpp56 was grown aerobically in Zobell marine broth 2216 (HiMedia Ltd., India) at 30°C under shaking conditions (120 rpm) for 24 h. The bacterial suspension was then pelleted by centrifugation and taken for genomic DNA (gDNA) extraction using a standard phenol-chloroform method as described earlier (9). The quality of the extracted gDNA was evaluated by running it in 0.8% agarose gel in 1× Tris-acetate-EDTA (TAE) buffer. After extraction, an aliquot of 1 ng DNA was enzymatically fragmented and tagged at both ends with adapters using a Nextera XT DNA library preparation kit (Illumina, San Diego, CA). The prepared library was purified using AMPure XP beads (Beckman Coulter, Inc., USA), enriched by PCR, and sequenced on an Illumina HiSeq sequencing platform (Illumina), producing 150-bp paired-end reads.

The entire whole-genome sequencing of *P. tetraodonis* strain kknpp56 produced 14,623,044 paired-end reads. The quality of the obtained reads was evaluated using FastQC software (10), and high-quality reads (>100 bp length and an average Phred score of >30) were *de novo* assembled into 115 contigs using MaSuRCA version 4.0.3. These contigs span 3,726,361 bp with a G+C content of 41%. The assembled contigs were further annotated using the NCBI Prokaryotic Genome Annotation Pipeline, predicting a total of 3,401 protein coding sequences (CDSs), 9 rRNA coding genes, and 137 tRNA coding genes, as well as 1 transfer-messenger RNA (tmRNA) coding gene. For all analyses, default parameters were used unless otherwise specified. Intriguingly, a number of genes, such as *csgA*, *csgE*, *csgF*, and *csgG*, involved in bacterial adhesion to the surfaces were identified in the investigated *P. tetraodonis* strain. The genome analysis of *P. tetraodonis* presented here provides scientific insights into the genes and cellular pathways involved in EPS biosynthesis of potent biofilm-forming marine bacteria as well as to understand the molecular basis supporting the genus *Pseudoalteromonas* to organize a surface-associated lifestyle in the marine environment.

### Data availability.

The whole-genome shotgun sequencing project has been deposited at DDBJ/ENA/GenBank under the accession number JAHFWE000000000. The version described in this paper is version JAHFWE000000000. The raw data were deposited in the NCBI database under BioProject number PRJNA732168.

## References

[1] Holmström C, Egan S, Franks A, McCloy S, Kjelleberg S. 2002. Antifouling activities expressed by marine surface associated *Pseudoalteromonas* species. FEMS Microbiol Ecol 41:47–58. doi:10.1111/j.1574-6941.2002.tb00965.x. 19709238

[2] Skovhus TL, Holmström C, Kjelleberg S, Dahllöf I. 2007. Molecular investigation of the distribution, abundance and diversity of the genus *Pseudoalteromonas* in marine samples. FEMS Microbiol Ecol 61:348–361. doi:10.1111/j.1574-6941.2007.00339.x. 17573938

[3] Bowman JP. 2007. Bioactive compound synthetic capacity and ecological significance of marine bacterial genus *Pseudoalteromonas*. Mar Drugs 5:220–241. doi:10.3390/md504220. 18463726 PMC2365693

[4] Thomas T, Evans FF, Schleheck D, Mai-Prochnow A, Burke C, Penesyan A, Dalisay DS, Stelzer-Braid S, Saunders N, Johnson J, Ferriera S, Kjelleberg S, Egan S. 2008. Analysis of the *Pseudoalteromonas tunicata* genome reveals properties of a surface-associated life style in the marine environment. PLoS One 3:e3252. doi:10.1371/journal.pone.0003252. 18813346 PMC2536512

[5] Tang BL, Yang J, Chen XL, Wang P, Zhao HL, Su HN, Li CY, Yu Y, Zhong S, Wang L, Lidbury I, Ding H, Wang M, McMinn A, Zhang XY, Chen Y, Zhang YZ. 2020. A predator-prey interaction between a marine *Pseudoalteromonas sp.* and Gram-positive bacteria. Nat Commun 11:285. doi:10.1038/s41467-019-14133-x. 31941905 PMC6962226

[6] Saravanan P, Nancharaiah YV, Venugopalan VP, Rao TS, Jayachandran S. 2006. Biofilm formation by *Pseudoalteromonas ruthenica* and its removal by chlorine. Biofouling 22:371–381. doi:10.1080/08927010601029103. 17178570

[7] Rajeev M, Sushmitha TJ, Toleti SR, Pandian SK. 2019. Culture dependent and independent analysis and appraisal of early stage biofilm-forming bacterial community composition in the Southern coastal seawater of India. Sci Total Environ 666:308–320. doi:10.1016/j.scitotenv.2019.02.171. 30798240

[8] Rajeev M, Sushmitha TJ, Prasath KG, Toleti SR, Pandian SK. 2020. Systematic assessment of chlorine tolerance mechanism in a potent biofilm-forming marine bacterium *Halomonas boliviensis*. Int Biodeterior Biodegradation 151:104967. doi:10.1016/j.ibiod.2020.104967.

[9] Sato MP, Ogura Y, Nakamura K, Nishida R, Gotoh Y, Hayashi M, Hisatsune J, Sugai M, Takehiko I, Hayashi T. 2019. Comparison of the sequencing bias of currently available library preparation kits for Illumina sequencing of bacterial genomes and metagenomes. DNA Res 26:391–398. doi:10.1093/dnares/dsz017. 31364694 PMC6796507

[10] Andrews S. 2010. FastQC: a quality control tool for high throughput sequence data. http://www.bioinformatics.babraham.ac.uk/projects/fastqc/.

